# Fungal Endocarditis: A Rare Case of Multiple Arterial Embolization

**DOI:** 10.7759/cureus.33312

**Published:** 2023-01-03

**Authors:** Francisca Beires, Raquel Rocha, Diana Mata, Sara Ramos, Nuno Moreno

**Affiliations:** 1 Internal Medicine, Hospital Pedro Hispano, Senhora da Hora, PRT; 2 Neurology, Hospital Pedro Hispano, Senhora da Hora, PRT; 3 Oncology, Instituto Português de Oncologia do Porto Francisco Gentil (FG) EPE, Matosinhos, PRT; 4 Cardiology, Hospital Pedro Hispano, Senhora da Hora, PRT

**Keywords:** surgical aortic valve replacement (savr), candida parapsilosis, antifungal medications, adult cardiac surgery, cerebral abscess, invasive fungal infections, infective endocarditis complications

## Abstract

Fungal endocarditis is a rare and fatal condition, with a mortality of up to 75%, affecting immunocompromised hosts with a predisposing condition, namely, a history of previous cardiac or noncardiac surgery. Embolization is frequent, accounting for 44% of cases, and as the most common site is the brain, it can cause leptomeningitis, parenchymal granulomas, or abscesses. This case report describes a man with aortic valve replacement one year ago and a recent carotid endarterectomy who was admitted with fever and neurological deficits. The workup permitted a diagnosis of fungal endocarditis, and the patient underwent a combined and aggressive treatment approach with antifungal therapy and surgery, with a successful replacement of the aortic valve. During hospitalization, the patient’s neurological status deteriorated, and a cerebral abscess was discovered on the left frontal lobe. Despite the poor prognosis, the patient recovered slowly and was discharged from the hospital three months later. The present case highlights the high index of suspicion needed for the diagnosis and the need for a multidisciplinary team to approach these patients to achieve a positive outcome.

## Introduction

Fungal endocarditis (FE) is a rare and fatal condition, with an incidence of 2-4% and a mortality rate of up to 75% [1]. The disease can present as native valve endocarditis, prosthetic valve endocarditis, endocardial surface inflammation, and cardiac device-related infective endocarditis, with the aortic valve being the most commonly affected (44%) [1]. Immunocompromised hosts, patients with previous cardiac or noncardiac surgery, long courses of antibiotics, and indwelling devices are at risk. The diagnosis is challenging because of exceedingly varied and nonspecific manifestations, such as fever, heart murmurs, heart failure, major peripheral embolization, and focal or generalized neurological features [2]. The most commonly isolated fungus is *Candida albicans*, followed by other *Candida* and *Aspergillus* species [2]. The most common site of embolization is cerebral; however, neurocandidiasis is rare (18-52% of disseminated candidiasis) and usually presents with meningoencephalitis or intracranial abscesses, either as an isolated phenomenon or associated with meningitis [3]. The abscesses are usually small, multiple, and associated with disseminated infections and with a mortality of 85-100% [3-5].

To the best of our knowledge, it is the second paper that reports a good outcome in a patient* *with endocarditis by *Candida parapsilosis* and multiple embolizations, including a cerebral abscess [6].

## Case presentation

**Figure 1:**
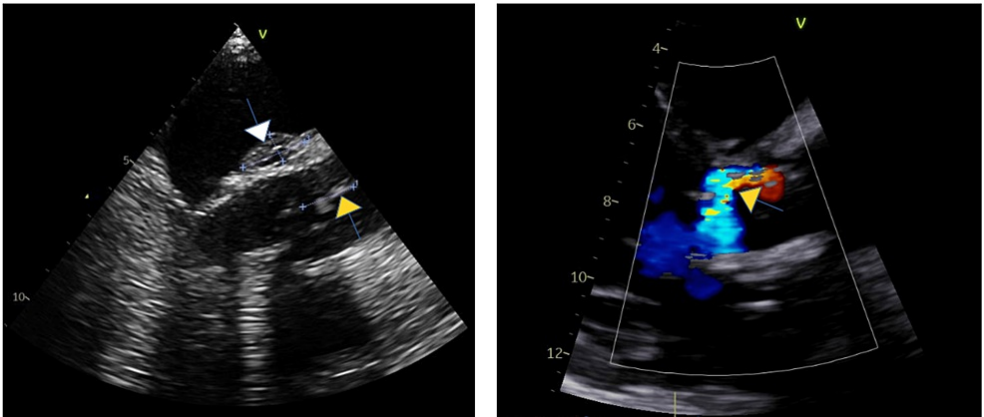
Transthoracic echocardiogram (TTE) In the left image, TTE shows a biological aortic valve with vegetation of 19 mm (yellow arrow) and an abscess on the mitral-aortic intervalvular fibrosa measuring 19x10 mm (white arrow). In the right image, TTE after biological valve replacement shows moderate intra and periprosthetic dysfunction on the aortic valve (yellow arrow).

A 70-year-old man was admitted with frontal headaches, dysarthria, balance deficits, and vertigo starting three hours prior. His past medical history revealed hypertension, dyslipidemia, type 2 diabetes, and valvular and ischemic cardiopathy, submitted to biological aortic valve replacement the year before without valvular dysfunction and preserved left ventricular ejection fraction. The patient also had a prior atherosclerotic disease, namely, right carotid stenosis, subjected to endarterectomy three months before. The patient mentioned an episode one month prior to admission, with fever and shivering where, after collecting blood cultures, he was discharged. On admission to the emergency room, he scored four on the National Institutes of Health Stroke Scale (NIHSS). Brain computed tomography (CT) revealed a posterior circulation stroke in the right paramedian cerebellum and an irregularity in the right carotid artery without significant restenosis. The patient underwent magnetic resonance imaging (MRI), which showed additional ischemic lesions in the anterior and posterior vascular territories of both hemispheres and raised the possibility of embolic etiology. The patient was maintained on anticoagulation and monitored with no documented arrhythmic events. Transthoracic echocardiogram (TTE) showed no valvular dysfunction or vegetation. During the first seven days of hospitalization, the patient was persistently subfebrile, with an elevated C-reactive protein level of 115 mg/L and sedimentation rate of 78 mm/1st hour, prompting the collection of blood cultures, urine culture, and a lumbar puncture. The cerebral spinal fluid showed elevated protein concentration (62.1 mg/dL) with pleocytosis (10 cells), urine cultures were negative, and, after several days, *Candida parapsilosis* was isolated in all sets of hemocultures. Therefore, micafungin treatment was initiated immediately. Due to the likelihood of endocarditis, the patient underwent transesophageal echocardiography (TEE), which showed a pediculated mass of 17 mm in the arterial phase of the non-coronary cuspid and an abscess on the mitral-aortic intervalvular fibrosa measuring 19 × 10 mm (Figure 1).

At this point, the diagnosis of FE was reached with two major Duke criteria, namely, positive blood cultures and echocardiogram showing valvular vegetation (Table 1) [7].

**Table 1:** Modified Duke criteria for infective endocarditis

Major Criteria	Minor Criteria
Positive blood cultures for typical microorganisms	Fever (>38^o^C)
Echocardiogram showing valvular vegetation	Predisposing cardiac lesion
	Intravenous drug abuse
	Embolic phenomena
	Immunological phenomena
	Microbiological evidence for atypical microorganisms
Definite Infective Endocarditis: 2 major criteria or 1 major + 3 minor criteria	
Probable Infective Endocarditis: 1 major + 1 minor criteria or 3 minor criteria	

**Figure 2:**
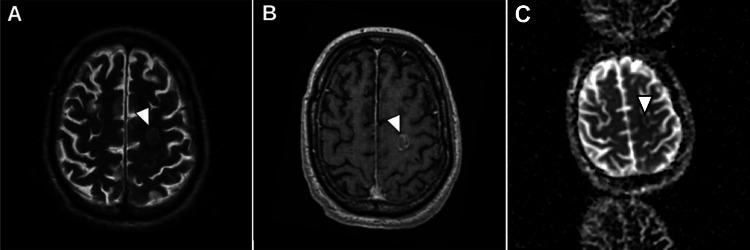
Cerebral magnetic resonance images Images A and B: Arrowheads pointing to a cortical lesion on the superior left frontal gyrus with a hypersignal in the T2 sequence with peripheral enhancement. Image C: Arrowhead pointing to a cortical lesion on the superior left frontal gyrus with restricted diffusion.

The patient underwent magnetic angiography resonance imaging and cerebrovascular ultrasound to verify the lesion in the right carotid artery, proving it to be a site of embolization. A full-body CT scan excluded other embolization sites. Two weeks later, the patient developed fever and psychomotor retardation, which prompted another lumbar puncture showing increased pleocytosis (47 cells). Assuming neurocandidiasis, antifungal therapy was changed to flucytosine and amphotericin because of the penetration of the blood-brain barrier, which the patient completed six days prior to his transfer to another hospital for aortic biologic valve replacement. Antifungal therapy was not changed before due to clinical improvement of the patient and decrease in inflammatory parameters. Implantation of the valve was particularly difficult due to the dissection of the mitral-aortic fibrosis. This led to a controlled TTE ascertaining a dysfunction of the valve with moderate intra- and periprosthetic dysfunction with a maintained biventricular ejection fraction. The patient remained stable and maintained antifungal therapy; however, due to fluctuating neurological deficits, a cerebral MRI was repeated, showing a 17 × 13 × 10 mm abscess on the left frontal (Figure 2).

The case was discussed in a multidisciplinary team meeting with Neurology, Neurosurgery, Cardiology, Cardiothoracic Surgery, Internal Medicine, and Infectious Diseases. At this point, the patient had no indication of further intervention either on the aortic valve or on the right carotid artery, due to an active uncontrolled infectious illness with a very poor prognosis. During hospitalization, the patient improved while continually undergoing antifungal therapy, completing 65 days with sterile hemocultures and the complete disappearance of the cerebral abscess as well as the vegetation on the carotid artery. The patient was discharged under lifelong fluconazole treatment.

## Discussion

FE is a misdiagnosed entity because of the heterogeneity and non-specificity of symptoms such as dyspnea, thoracic pain, asthenia, fever, altered mental status, new cardiac murmur, and acute or chronic heart failure. Peripheral embolization is common in 44% of FE cases, with brain embolization being the most common [8]. Fungal cerebritis is the earliest manifestation of brain infection and the precursor of abscess development. The most common pathogens are *Cryptococcus, Aspergillus*, and *Candida*. Brain abscesses have an estimated mortality of 85-100% and are more likely to be multiple and involve the basal ganglia, whereas bacterial abscesses are often solitary lesions sparing the basal ganglia [4-5]. With regards to the agent, *Candida albicans* is the most common pathogen followed by *Candida parapsilosis*, which was earlier considered to be a non-pathogenic strain until 1940. Some of the common predisposing factors for *C. parapsilosis* include a long stay in the intensive care unit, prosthetic valves, IV drug use, parenteral nutrition, abdominal surgery, immunosuppression, treatment with broad-spectrum antibiotics, previous valvular disease, and organ failure [9]. Our patient had a history of diabetes mellitus, aortic valve replacement, and a recent carotid endarterectomy. Diagnosis of FE is extremely difficult because fungi are fastidious microorganisms that take at least five days to appear in hemocultures. In addition, the sensitivity of hemocultures is far from admissible, accounting for about 50%. Non-cultural tests are emerging as a means of improving diagnostic ability and patient care. Mannan and anti-Mannan immunoglobulin G (IgG) antibodies' use combined have a sensitivity of up to 83% and a specificity of 86%, mainly for *Candida* species other than *C. parapsilosis*. 1,3-β-d-glucan, a component of the fungal wall, has a sensitivity of 75-80% and specificity of 80%, but it is nonspecific to the type of fungus. Other tests, such as polymerase chain reaction (PCR) or the T2-*Candida* Panel, seem to be good tools for the diagnosis of candidemia [10]. Treatment should always include surgery because of the difficulty in effectively eradicating these pathogens using antifungal therapy alone. A regimen combining amphotericin B +/- flucytosine is recommended for at least seven days, followed by valvular replacement surgery, as per European Society of Clinical Microbiology and Infectious Diseases (ESCMID) guidelines [11]. Long-term suppressive fluconazole therapy is recommended, but its duration remains debatable. In our patient, the first approach with echinocandin was apparently effective, reducing subfebrile episodes and decreasing inflammatory parameters; however, dissemination of the disease was documented afterward. It remains unknown whether the major contributor to the formation of the cerebral abscess was the choice of antifungal treatment or the dissemination of the disease during valve replacement surgery. Hence, this case raises significant learning points about the timing of the surgery - in our patient, three weeks after documenting sterile hemocultures, and about the initial choice of antifungal therapy. Perhaps the use of amphotericin and flucytosine as first-line antifungal therapy would have decreased the duration of hospitalization and the complications, namely, the formation of the cerebral abscess and the dissection of the mitral-aortic intervalvular. Despite the use of a combination of medical and surgical therapies, the mortality rate associated with *C. parapsilosis* endocarditis remains as high as 40%. Poor outcome factors include older age, heart failure at baseline, persistent candidemia, nosocomial acquisition, heart failure as a complication, and intracardiac abscess. Follow-up of these patients is of utmost importance due to the recurrence rate of FE in 30% to 40% of patients, which can occur up to nine years later [2].

## Conclusions

The aim of this case report is to alert clinicians to FE, its high heterogeneity of symptoms, and its mutation ability with more embolic and immunological phenomena when compared to bacterial endocarditis. The diagnosis of FE is even more challenging than that of bacterial endocarditis, requiring repeated blood cultures and serial TTE and TEE. Also, the choice of antifungal therapy is important and must consider sites of embolization. The mortality of this disease is extremely high because it primarily affects patients with an immunocompromised status and causes high morbidity, with recurrent hospital admissions and progressively degrading performance status. Therefore, early detection and aggressive surgical and medical treatment are of utmost importance for good patient outcomes.
